# Development of JAK2 positive myelofibrosis on a background of chronic myeloid leukaemia

**DOI:** 10.1002/jha2.453

**Published:** 2022-08-03

**Authors:** Christopher Gerard Mullen, Victoria Louise Campbell, Tamasin Doig, Alice Klauser

**Affiliations:** ^1^ Department of Haematology Royal Infirmary of Edinburgh, Little France Crescent Edinburgh UK; ^2^ Department of Haematology Western General Hospital Edinburgh UK; ^3^ Department of Histopathology Western General Hospital Edinburgh UK

1

A 50‐year‐old male patient was admitted with a history of constitutional symptoms and weight loss with a marked leucocytosis (WBC 270 × 10^9^/L) and blood film appearances suggestive of chronic myeloid leukaemia (CML). Platelet count was normal at presentation (298 × 10^9^/L).

*BCR‐ABL1* was detected (b2a2) confirming CML. Bone marrow trephine sections revealed a markedly expanded myeloid series but no excess of blasts (Figure 1). Megakaryocytes were noted to be reduced in size with some bare nuclei seen. Cytogenetics confirmed 46,XY, t(9;22)(q34;q11) with no additional anomalies. He was commenced on imatinib but developed progressive full blood count abnormalities (WBC 9.6 × 10^9^/L, Neutrophils 6.7 × 10^9^/L, Hb 99 g/L, Platelets 650 × 10^9^/L) with an increase in his *BCR‐ABL1* ratio 3 months following this.

**FIGURE 1:**
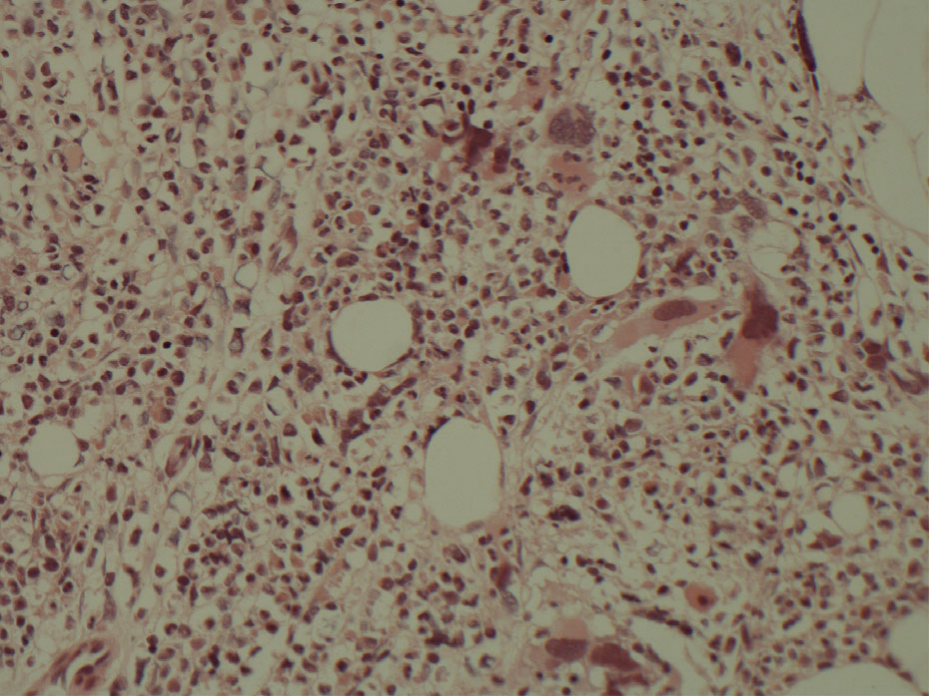
Bone marrow trephine section at diagnosis, H&E, 20x objective lens

He was switched to nilotinib and had a further bone marrow evaluation, which revealed a marked increase in reticulin fibres (MF‐2) but no evidence progression to accelerated or blast phase CML.

Despite an excellent initial response to nilotinib (achieving molecular response (MR‐2) after 7 weeks), he developed a persistent neutrophilia and thrombocytosis.

Due to his blood count abnormalities and bone marrow fibrosis, *JAK2* testing was performed and revealed the V617F mutation. A repeat bone marrow examination (to assess cytogenetic response) revealed increased megakaryopoiesis with dysplastic, spindle‐shaped, and hyperlobulated nuclei, with clustering, with increased reticulin fibres (MF‐2 with some focal areas of MF‐3) noted again (Figures 2 and 3). No *ABL* kinase domain mutations were detected at time of *JAK* testing.

**FIGURE 2:**
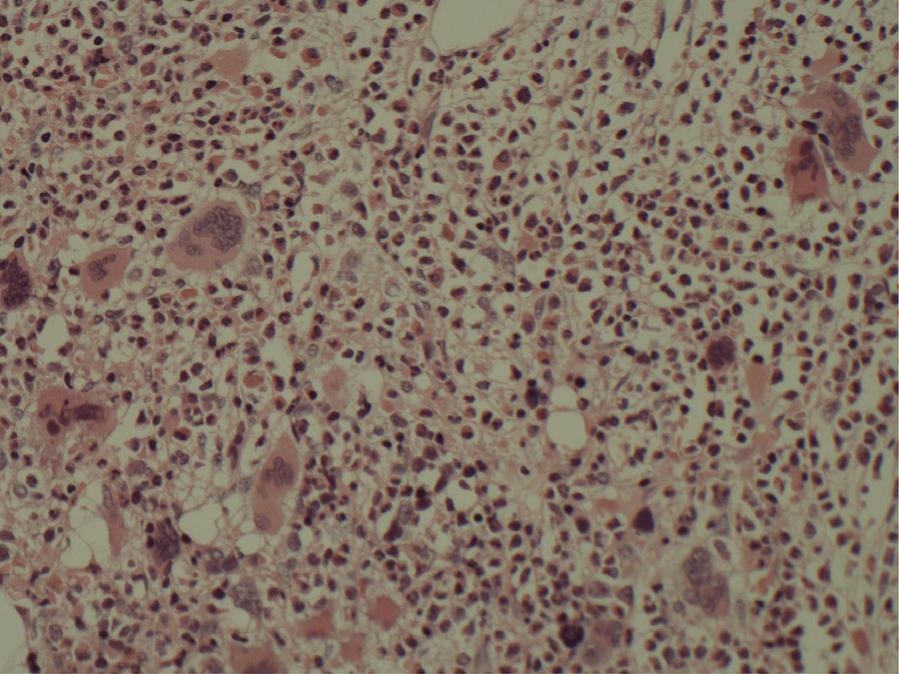
Bone marrow trephine section repeat, H&E, 20x objective lens

**FIGURE 3:**
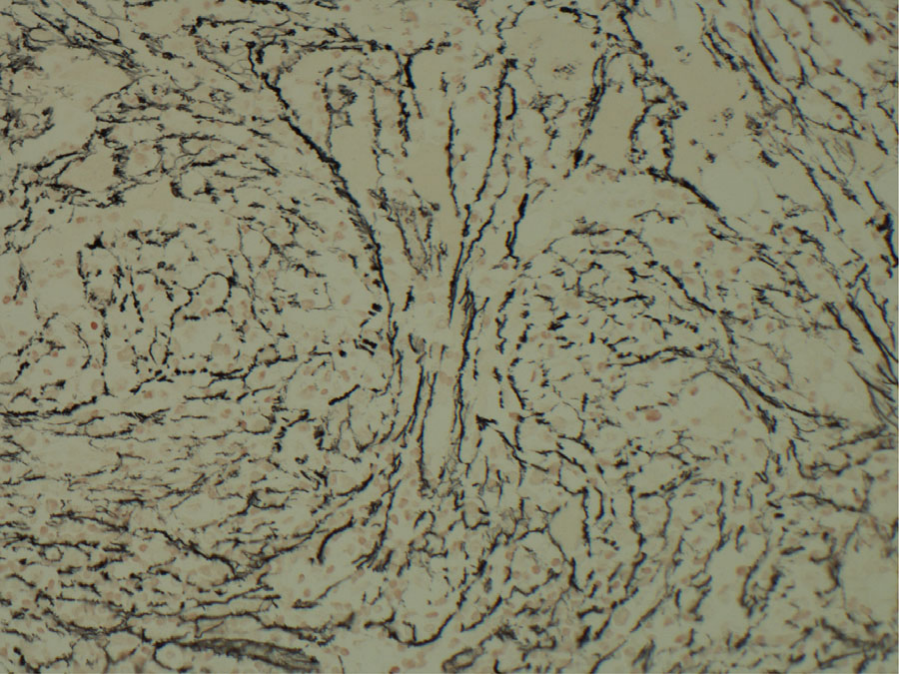
Bone marrow trephine section repeat, reticulin, 20x objective lens

He was diagnosed with *JAK2* positive myelofibrosis (independent of his known CML) (DIPSS score 0, associated with medial survival of over 14 years). The patient began to experience headaches and was commenced on hydroxycarbamide 500 mg once daily in case these were vaso‐occlusive in origin. He remains well and in a good molecular *BCR‐ABL1* response on both therapies over 2 years following the diagnosis. He achieved major molecular remission (MR4) at 26 months.

Fibrosis within the bone marrow in CML patients is common. The presence of both *BCR‐ABL1* and *JAK2* V617F mutations is highly unusual. It is unclear whether these mutations arose within the same clone or within differing clones. Previous cases have reported unmasking of Philadelphia negative myeloproliferative neoplasms during treatment for CML [1]. Of note, retrospective testing on the initial bone marrow samples from the CML was first diagnosed for *JAK2* V617F was negative.

## CONFLICT OF INTEREST

The authors report no conflict of interest.

## AUTHOR CONTRIBUTIONS

Christopher Gerard Mullen wrote the clinical summary. Tamasin Doig provided images of bone marrow trephine sections. Victoria Louise Campbell and Alice Klauser made revisions and edits to the clinical summary.

## FUNDING INFORMATION

None.

## ETHICS STATEMENT

This article involved no human or animal research participants.

## PATIENT CONSENT

No patient identifiable information was submitted.

2

## Data Availability

Data sharing is not applicable to this article as no datasets were generated or analyzed during the current study.
